# Inflammatory profile of incident cases of late-onset compared with young-onset rheumatoid arthritis: A nested cohort study

**DOI:** 10.3389/fmed.2022.1016159

**Published:** 2022-11-08

**Authors:** Natalia Mena-Vázquez, Jose Manuel Lisbona-Montañez, Rocío Redondo-Rodriguez, Arkaitz Mucientes, Sara Manrique-Arija, José Rioja, Aimara Garcia-Studer, Fernando Ortiz-Márquez, Laura Cano-García, Antonio Fernández-Nebro

**Affiliations:** ^1^Instituto de Investigación Biomédica de Málaga (IBIMA)-Plataforma Bionand, Málaga, Spain; ^2^Unidad de Gestión Clínica (UGC) de Reumatología, Hospital Regional Universitario de Málaga, Málaga, Spain; ^3^Departamento de Medicina y Dermatología, Universidad de Málaga, Málaga, Spain

**Keywords:** late-onset rheumatoid arthritis, young-onset rheumatoid arthritis, aging, inflammation, early rheumatoid arthritis

## Abstract

**Objectives:**

To describe the characteristics of patients between late-onset rheumatoid arthritis (LORA) with young-onset (YORA), and analyze their association with cumulative inflammatory burden.

**Methods:**

We performed a nested cohort study in a prospective cohort comprising 110 patients with rheumatoid arthritis (RA) and 110 age- and sex-matched controls. The main variable was cumulative inflammatory activity according to the 28-joint Disease Activity Score with erythrocyte sedimentation rate (DAS28-ESR). High activity was defined as DAS28 ≥ 3.2 and low activity as DAS28 < 3.2. The other variables recorded were inflammatory cytokines, physical function, and comorbid conditions. Two multivariate models were run to identify factors associated with cumulative inflammatory activity.

**Results:**

A total of 22/110 patients (20%) met the criteria for LORA (≥ 60 years). Patients with LORA more frequently had comorbid conditions than patients with YORA and controls. Compared with YORA patients, more LORA patients had cumulative high inflammatory activity from onset [13 (59%) vs. 28 (31%); *p* = 0.018] and high values for CRP (*p* = 0.039) and IL-6 (*p* = 0.045). Cumulative high inflammatory activity in patients with RA was associated with LORA [OR (95% CI) 4.69 (1.49–10.71); *p* = 0.008], smoking [OR (95% CI) 2.07 (1.13–3.78); *p* = 0.017], anti–citrullinated peptide antibody [OR (95% CI) 3.24 (1.15–9.13); *p* = 0.025], average Health Assessment Questionnaire (HAQ) score [OR (95% CI) 2.09 (1.03–14.23); *p* = 0.034], and physical activity [OR (95% CI) 0.99 (0.99–0.99); *p* = 0.010]. The second model revealed similar associations with inflammatory activity in patients with LORA.

**Conclusion:**

Control of inflammation after diagnosis is poorer and comorbidity more frequent in patients with LORA than in YORA patients and healthy controls.

## Introduction

Rheumatoid arthritis (RA) is an immune-mediated disease characterized by chronic synovitis, joint destruction, and functional disability. It can affect adults of all ages, although its incidence increases with age, peaking at 50–60 years in Spain (1). While the incidence rate of RA is lower in persons aged more than 60 years, it continues to be high, and the characteristics of the disease can differ according to the patient (2–4).

The World Health Organization defines an older person as one whose chronological age is 60 years or more.^1^ Improvements in life expectancy in recent years mean that the percentage of older persons with RA is increasing, with this population accounting for 10–33% of all cases (5). Previous studies report that the clinical presentation, severity, and prognosis of RA can vary according to age at diagnosis (2, 3, 5–7). Constitutional syndrome and other comorbid conditions such as chronic anemia and kidney failure are also more frequently late in onset (7), whereas manifestations such as Sjögren syndrome, interstitial lung disease (2, 8), and findings for seropositive results are controversial (9).

Older patients with inflammatory diseases have poorer disease-related health outcomes and concomitant conditions (10, 11). Furthermore, studies on disease severity are inconsistent, with some authors suggesting that patients with late-onset rheumatoid arthritis (LORA) could be less likely to achieve complete remission (6, 12, 13). Some studies have associated this greater severity with increased inflammatory activity and higher acute phase reactant values than in patients whose disease first manifests at a younger age (2, 6, 14). This group of patients has also been reported to less frequently receive disease-modifying antirheumatic drugs (DMARDs)—both conventional synthetic DMARDs (csDMARDs) and targeted synthetic DMARDs (tsDMARDs), as well as biologic DMARDS (bDMARDs)—or lower doses than desired, perhaps owing to the increased risk of toxicity, cardiovascular events, and cancer (15–18). However, no studies compare cumulative inflammatory activity over time after diagnosis based on activity indexes and inflammatory cytokines between patients with LORA and those with early onset disease and healthy controls. Consequently, the main objectives of our study were to describe cumulative inflammatory activity after diagnosis of the disease in patients with LORA and to identify the role of age of onset and other severity-related factors associated with poorer outcome.

## Patients and methods

### Study design and participants

This is a nested cohort study with 110 patients with RA (≥ 16 years) from a cohort of incident cases recruited between 2007 and 2011 and prospectively followed at the Department of Rheumatology of Hospital Universitario Regional de Málaga, Málaga, Spain. Patients with RA were classified according to the 2010 criteria of the American College of Rheumatology/European League Against Rheumatism (19). All the patients were diagnosed and treated during the first 12 months after onset of their disease. Patients were stratified by age at diagnosis of RA [young-onset rheumatoid arthritis (YORA), < 60 years; LORA, ≥ 60 years] (2). Similarly, we recruited 110 age- and sex-matched controls from among persons without rheumatic disease attending primary care centers in the same health district. The controls were also stratified by age for matching with patients diagnosed with LORA. Controls with an associated inflammatory disease were excluded. Patients with RA and an inflammatory or rheumatic disease other than RA (except secondary Sjögren syndrome) were also excluded. The study was approved by the Local Medical Ethics Committee, and all the patients gave their written informed consent before inclusion (Project identification code 4/2016, P19).

### Protocol

After signing the informed consent document, all participants were interviewed and examined by a rheumatologist at the index date, which was defined as the date of the last observation of the patients in the cohort, and the date of the recruitment of controls. All clinical and laboratory data were collected. Patients with RA are usually assessed in the outpatient clinic every 3–6 months using a pre-established questionnaire to collect data on inflammatory activity and physical function. We collected data on inflammatory activity and physical function throughout follow-up. Blood samples were collected after a 12–16-h fast and before 10:00 a.m.

### Evaluation of inflammatory activity and physical function

The main variables were inflammatory activity at the index date and cumulative activity over time at the visits following inclusion in the cohort. The cumulative activity was calculated as the arithmetic mean of all the values recorded regularly from diagnosis to date index (during follow-up). Inflammatory activity was measured using the 28-joint Disease Activity Score for rheumatoid arthritis with erythrocyte sedimentation rate (DAS28-ESR) (range 0–9.4) (20). DAS28-ESR > 3.2 was considered high activity and ≤ 3.2 low activity. Other inflammation-related variables were blood levels of C-reactive protein (CRP, mg/dL) and ESR (mm/h). We also evaluated inflammatory activity using serum levels of interleukin (IL) 6, IL-1β, insulin-like growth factor 1 (IGF 1), and anti-LDL oxidase based on the chemiluminescent enzyme assay (QuantiGlo^®^). Tumor necrosis factor alpha (TNF-α) was determined using automated immunoassay (Immulite^®^, Diagnostic Products Corporation, Los Angeles, CA, USA). Physical function at the index date and the mean during follow-up was measured using the Health Assessment Questionnaire (HAQ) (21).

### Other variables

We also studied demographic, clinical, anthropometric, and treatment-related variables. Demographic and clinical variables included age (years), sex (male/female), and comorbidities associated with traditional cardiovascular risk factors (smoking, obesity, arterial hypertension, diabetes mellitus, history of cardiovascular disease, and sedentary lifestyle), comorbidities included in the Charlson index, and other comorbidities not covered above, such as fibromyalgia, thyroid disease, osteoporosis, and Sjögren syndrome. Mediterranean diet and sedentary lifestyle were assessed using a validated questionnaire on adherence to the Mediterranean diet (MEDAS) (22), which consists of 14 items (patients answering affirmatively to 9 or more items are considered to adhere, whereas those answering to fewer than 9 items are not), and the International Physical Activity Questionnaire (IPAQ) (23), whose results are expressed as metabolic equivalent of task (MET). A MET is the metabolic rate at rest, i.e., the amount of oxygen consumed by a person who is sitting at rest (24). The level of physical activity is considered low/sedentary or insufficient for meeting recommendations on healthy activity (<600 MET-minutes in the previous week) or moderate/high or sufficient for achieving a moderate level of activity (> 600 MET-minutes during the previous week). The anthropometric parameter assessed was body mass index [BMI (kg/height in m^2^)]. Patients were classified as underweight (< 18.5), normal weight (18.5–24.9), overweight (25–29.9), and obese (> 30), according to the definitions of the World Health Organization (WHO) (25).

The characteristics of patients with RA included date of onset of the disease, disease duration (from diagnosis to index date), and diagnostic delay (months from onset of symptoms to diagnosis). We recorded the presence of antibodies and their titers [rheumatoid factor, positive if > 10 Iμ/mL; anti–citrullinated peptide antibody (ACPA), positive if > 20 Iμ/mL] and the presence or absence of radiologic erosions. We recorded all drugs taken until the index date, including csDMARDs (methotrexate, leflunomide, and sulfasalazine), bDMARDs [tumor necrosis factor inhibitors (anti-TNF), tocilizumab, abatacept, rituximab, and ustekinumab], tsDMARDs [Janus kinase inhibitors (JAKi), such as tofacitinib and baricitinib], and corticosteroids.

### Statistical analysis

A descriptive analysis was made of epidemiological characteristics and comorbid conditions for patients and controls, as was an analysis of clinical-laboratory characteristics for patients with RA according to age at diagnosis (≥ 60 vs. < 60 years). Qualitative variables were expressed as absolute number and percentage, and quantitative variables were expressed as mean and standard deviation (SD) or median and interquartile range (IQR), according to whether the variables were distributed normally or not normally (Kolmogorov-Smirnov test). The χ^2^- and *t*-test or Mann-Whitney test were used to compare the main characteristics between patients who had LORA and those who did not and between patients and controls. We constructed a receiver operating characteristic (ROC) curve with area under ROC curve (AUC) for associations between cumulative inflammatory activity (DAS28-ESR) and RA according to age at diagnosis. Finally, two multivariate logistic regression models were run (dependent variable: high inflammatory activity). The first aimed to identify independent variables in patients with RA. The second was run for patients with LORA. The multicollinearity of independent variables was checked using Pearson correlation coefficient. If the r-coefficient was > 0.4, we entered the variables separately in the models, to see if any change either in associations with the dependent variable or in the explaining value of the model had occurred. Sample size was calculated based on an alpha risk of 0.15 and a beta risk of 0.2 in a 2-sided contrast. A total of 53 patients were necessary to identify differences in inflammatory activity between patients with LORA and patients with YORA, and a total of 20 patients were necessary in each group in order to detect differences in physical function (2). Statistical significance was set at *p* < 0.05 for all the analyses, which were performed using R 2.4–0.

## Results

**FIGURE 1:**
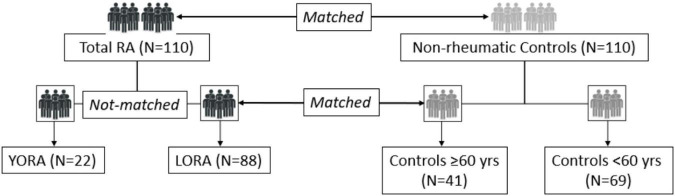
Patient flow chart.

Between June 2017 and September 2020, we consecutively recruited 110 patients with RA and 110 controls. Most participants were women (80%), with a mean (SD) age at the index date of 48.9 (11.3) years. Age was higher in men than in women [53.0 (10.0) vs. 47.8 (11.4); *p* = 0.048]. A total of 22/110 patients (20%) fulfilled the criteria for LORA (≥ 60 years); of the 110 controls included, 41 (37.2%) were included after age ≥ 60 years. Figure 1 shows the flow chart for patients and controls.

### Epidemiologic and clinical characteristics and comorbidities

Table 1 shows the clinical-epidemiologic characteristics at the index date of patients with LORA and YORA. The comparison between patients and non-rheumatic controls aged ≥ 60 years at the index date are described in Supplementary Table 1.

**TABLE 1:** Clinical-epidemiologic characteristics of patients with LORA and YORA.

	LORA *N* = 22	YORA *N* = 88	*P*-value
**Epidemiologic characteristics**			
Age in years, mean (SD)	69.2 (4.6)	52.5 (6.3)	<0.001
Female sex; *n* (%)	14 (63.6)	74 (84.1)	0.032
Caucasian race, *n* (%)	22 (100.0)	86 (97.7)	0.475
BMI (kg/m^2^), mean (SD)	29.8 (5.3)	28.0 (5.0)	0.151
Smoking history			0.690
Never smoked, *n* (%)	10 (45.5)	37 (42.0)	
Ex-smoker, *n* (%)	7 (31.8)	23 (26.1)	
Active smoker, *n* (%)	5 (22.7)	28 (31.8)	
**Comorbidities**			
Arterial hypertension, *n* (%)	11 (50.0)	17 (19.3)	0.003
Diabetes mellitus, *n* (%)	2 (9.1)	4 (4.5)	0.401
Dyslipidemia, *n* (%)	8 (36.4)	17 (19.3)	0.088
Obesity (WHO) (BMI ≥ 30), *n* (%)	10 (45.5)	28 (31.8)	0.229
Cardiovascular disease, *n* (%)	6 (27.3)	12 (13.6)	0.122
Sjögren syndrome, *n* (%)	3 (13.6)	12 (13.6)	1.000
Osteoporosis, *n* (%)	7 (31.8)	12 (13.6)	0.044
**Clinical characteristics**			
Time since diagnosis of RA, months, median (IQR)	83.8 (76.3–101.9)	97.1 (78.1–129.2)	0.072
Diagnostic delay, months, median (IQR)	6.8 (4.6–11.3)	8.1 (4.5–13.0)	0.782
Erosions, *n* (%)	15 (68.2)	53 (60.2)	0.492
RF > 10, *n* (%)	18 (81.8)	72 (81.0)	1.000
ACPA > 20 U/ml, *n* (%)	18 (81.8)	70 (79.5)	0.812
High ACPA > 340 U/mL, *n* (%)	8 (36.4)	28 (31.8)	0.684
**Treatment**			
csDMARD, *n* (%)	20 (87.0)	82 (88.2)	0.873
Methotrexate, *n* (%)	16 (69.6)	63 (67.7)	0.867
Leflunomide, *n* (%)	3 (13.0)	9 (9.7)	0.635
Sulfasalazine, *n* (%)	2 (8.7)	11 (11.8)	0.670
Hydroxychloroquine, *n* (%)	0 (0.0)	9 (9.7)	0.126
bDMARD, *n* (%)	6 (26.1)	36 (38.7)	0.259
Anti-TNF-α, *n* (%)	5 (21.7)	27 (29.0)	0.483
Anti-IL-6, *n* (%)	1 (4.3)	6 (6.5)	0.704
Rituximab, *n* (%)	0 (0.0)	2 (2.2)	0.664
Tofacitinib, *n* (%)	0 (0.0)	1 (1.1)	0.175
Combination of csDMARD and bDMARD, *n* (%)	4 (17.4)	30 (32.3)	0.124
Corticosteroids at index date, *n* (%)	3 (13.0)	17 (18.3)	0.552
Dose of corticosteroids (mg/kg), median (IQR)	5.0 (2.5–5.0)	5.0 (5.0–6.2)	0.358

LORA, late-onset rheumatoid arthritis; YORA, young-onset rheumatoid arthritis; SD, standard deviation; IQR, interquartile range; BMI, body mass index; RF, rheumatoid factor; ACPA, anti–citrullinated peptide antibody; DMARD, disease-modifying antirheumatic drug; csDMARD, conventional synthetic DMARD; bDMARD, biologic DMARD.

Most patients with RA were women, and the mean age was approximately 56 years at the index date. The median (IQR) time since diagnosis of 93.2 (77.6–123.4) months. Somewhat more than half of the patients had been smokers or were actively smoking, and almost all patients had a positive serology result. All patients were receiving therapy with DMARDs: 102/110 (92.7%) with csDMARDs and 42/110 (38.2%) with bDMARDs, mostly anti-TNF agents. Only 1 patient with RA received a JAK inhibitor. Epidemiologic characteristics, frequency of autoantibodies, and treatments used were mostly similar in patients with LORA and YORA. However, LORA patients were more frequently male (*p* = 0.032), with a greater frequency of arterial hypertension (*p* = 0.003), osteoporosis (*p* = 0.044) (Table 1), kidney involvement (*p* = 0.045), liver involvement (*p* = 0.045), and history of cancer (*p* < 0.001) (Supplementary Table 2).

Furthermore, patients with LORA more frequently had a personal history of cardiovascular disease and osteoporosis, and 40% more patients smoked than controls aged ≥ 60 years.

### Study of inflammatory factors and cytokines in patients with rheumatoid arthritis according to age and controls

More than half of the patients with RA (62.7%) were in remission or had low disease activity at the index date. During follow-up, the mean DAS28-ESR value indicated that 71/110 patients (64.5%) had low disease activity, whereas 39 (35.5%) had high activity. As can be seen in Table 2, patients with LORA had higher DAS28-ESR scores at the index date and the mean during follow-up than patients with YORA. Similarly, patients with LORA had higher CRP values than YORA at the index date (*p* = 0.039) and IL-6 (*p* = 0.045) (Table 2). Also at diagnosis, patients with LORA had higher DAS28-ESR scores than patients with YORA [4.9 (1.1) vs. 4.4 (0.8); *p* = 0.040].

**TABLE 2:** Inflammatory factors and cytokines in patients with LORA and YORA.

	LORA *N* = 22	YORA *N* = 88	*P*-value
**Inflammatory activity**			
DAS28 average value, mean (SD)*	3.3 (1.0)	2.8 (1.0)	0.033
Remission-low activity, *n* (%)	9 (40.9)	62 (70.5)	0.010
Moderate-high activity, *n* (%)	13 (59.1)	26 (29.5)	0.010
DAS28 at index date, mean (SD)	3.1 (0.6)	2.9 (0.7)	0.078
Remission-low activity, n (%)	9 (40.9)	60 (68.2)	0.018
Moderate-high activity, *n* (%)	13 (59.1)	28 (31.8)	0.018
Average HAQ value, mean (SD)**	0.80 (0.7)	0.71 (0.4)	0.529
HAQ at index date, mean (SD)	0.79 (0.6)	0.76 (0.5)	0.729
**Laboratory parameters**			
ESR, mm/h, median (IQR)	19.5 (8.7–27.5)	13.0 (6.7–19.2)	0.102
Hemoglobin, g/dL, median (IQR)	13.1 (12.2–14.1)	13.9 (13.0–14.7)	0.034
Leukocytes, 10^9^/L, median (IQR)	6,735 (5,780–8,407)	6,295 (5,150–8,147)	0.258
Platelets, 10^9^/L, median (IQR)	236,000 (200,000–260,250)	243,000 (200,500–284,500)	0.239
Creatinine, mg/dL, median (IQR)	0.8 (0.7–0.9)	0.7 (0.6–0.8)	0.003
Total cholesterol (mg/dL), mean (SD)	208.9 (38.3)	199.0 (37.2)	0.271
LDL cholesterol (mg/dL), median (IQR)	119.0 (108.3–141.7)	113.0 (95.2–135.0)	0.222
HDL cholesterol (mg/dL), median (IQR)	57.5 (50.5–65.2)	59.5 (49.2–69.0)	0.208
Triglycerides (mg/dL), median (IQR)	108.0 (74.5–219.0)	84.5 (68.2–118.7)	0.025
Homocysteine, mg/L, median (IQR)	16.3 (7.0–34.0)	13.9 (11.6–16.0)	0.228
**Cytokines**			
IL6, pg/mL, median (IQR)	16.3 (7.0–34.0)	9.9 (5.2–17.6)	0.045
CRP, mg/L, mean (SD)	6.9 (4.1)	4.1 (3.7)	0.039
IL-1β, pg/mL, median (IQR)	4.3 (4.2–4.5)	4.3 (4.1–4.4)	0.660
TNF-α, pg/mL, median (IQR)	6.1 (3.6–22.4)	5.0 (3.7–22.4)	0.308
IGF-1, pg/mL, median (IQR)	109.0 (70.5–64.5)	182.5 (113.8–244.0)	0.112
LDL oxidase (U/L), median (IQR)	1.7 (0.4–5.3)	2.6 (0.7–5.7)	0.605
**Physical activity and Mediterranean diet**			
IPAQ, METs, median (IQR)	210 (165.0–922.5)	353.3 (198.0–890.7)	0.224
Sedentary lifestyle, *n* (%)	15 (68.2)	50 (56.8)	0.430
MEDAS (> 9), *n* (%)	10 (45.5)	56 (63.6)	0.119

LORA, late-onset rheumatoid arthritis; YORA, young-onset rheumatoid arthritis; DAS28, 28-joint Disease Activity Score; HAQ, Health Assessment Questionnaire; ESR: erythrocyte sedimentation rate; HDL, high-density lipoprotein; LDL, low-density lipoprotein; IL: interleukin; CRP, C-reactive protein; TNF, tumor necrosis factor; IGF, insulin-like growth factor; IPAQ, International Physical Activity Questionnaire; MEDAS, Validated questionnaire to determine adherence to a Mediterranean diet. *DAS28 average value, mean (SD), is the cumulative inflammatory activity calculated as the mean of DAS28-ESR during follow-up. **Average HAQ value, mean (SD), is the cumulative HAQ calculated as the mean of DAS28-ESR during follow-up.

Patients with LORA and controls aged ≥ 60 years generally had similar laboratory profiles, except for ESR, leukocytes, and homocysteine. Similarly, both groups of patients had higher levels of CRP, IL-6, IL-1β, and TNF-α than controls aged ≥ 60 years (Supplementary Table 3).

While no differences were found between the groups for adherence to a Mediterranean diet, patients with LORA were more sedentary than controls and engaged in less physical activity [median (IQR) = 210.0 (165.0–922.5) vs. 604.0 (214.0–990.0) METS; *p* = 0.038] (Supplementary Table 3).

### Factors associated with inflammatory activity in patients with rheumatoid arthritis

As shown in Supplementary Table 4, disease activity was high in 39 of the 110 patients with RA (35.5%); among these, more patients had LORA (*p* = 0.010), a history of smoking (*p* = 0.009), obesity (*p* = 0.048), dyslipidemia (*p* = 0.035), osteoporosis (*p* = 0.046), a sedentary lifestyle (*p* = 0.014), and poorer adherence to a Mediterranean diet (*p* = 0.018). As for clinical-laboratory parameters, patients with RA whose was a high inflammatory activity that of other patients more frequently had high ACPA titers (*p* = 0.002), poorer physical function according to the HAQ (*p* = 0.004), and higher levels of ESR (*p* = 0.019), CRP (*p* = 0.027), and IL-6 (*p* = 0.046), as well as lower hemoglobin values (*p* = 0.021). No differences were observed with respect to DMARDs and corticosteroids between the groups.

Table 3 shows the results of the multivariate logistic regression analysis (dependent variable: cumulative high inflammatory activity during follow-up) in 110 patients with RA to determine how late onset behaves with respect to other factors associated with inflammatory activity. As can be seen, LORA was the factor most closely associated with cumulative inflammatory activity of RA. In comparison, smoking (current or previous), high ACPA titers, and greater involvement of physical function (HAQ) were associated with a greater risk of inflammatory activity, whereas physical activity was the only factor that had a protective effect.

**TABLE 3:** Multivariate analysis of factors associated with cumulative inflammatory activity in all patients with RA.

Variable	Univariate OR (95% CI)	Multivariate OR (95% CI)	*P*-value
LORA	3.444 (1.312–9.045)	4.694 (1.497–10.717)	0.008
Age, years	1.038 (1.010–1.079)		
Female sex	0.590 (0.228–1.525)		
Current or previous smoking	3.108 (1.594–8.243)	2.075 (1.137–3.789)	0.017
High ACPA (> 340 U/mL)	3.618 (1.563–8.375)	3.249 (1.156–9.137)	0.025
Radiological erosions	1.381 (0.610–3.837)		
Average HAQ*	2.100 (1.149–3.837)	2.093 (1.035–4.231)	0.034
Body mass index (kg/m^2^)	1.105 (1.020–1.198)		
csDMARDs	0.909 (0.205–4.025)		
bDAMRDs	1.422 (0.640–3.160)		
Corticosteroids	0.525 (0.469–3.431)		
IL-6, pg/mL	1.004 (0.989–1.011)		
Il-1β, pg/mL	1.420 (0.870–2.318)		
TNF-α, pg/mL	1.001 (0.998–1.005)		
IPAQ, METs	0.998 (0.996–0.999)	0.998 (0.996–0.999)	0.010

The dependent variable is high inflammatory activity (DAS28-ESR ≥ 3.2). Nagelkerke *R*^2^ = 0.431. Variables included in the equation: sex, LORA, current/previous smoking, high ACPA (> 340 μ/mL), HAQ at index date, BMI, METs. LORA, late-onset rheumatoid arthritis; ACPA, anti–citrullinated peptide antibody; HAQ, Health Assessment Questionnaire; DMARD, disease-modifying antirheumatic drug; csDMARD, conventional synthetic DMARD; bDMARD, biologic DMARD; ESR, erythrocyte sedimentation rate; Il-6, interleukin 6; CRP, C-reactive protein; TNF, tumor necrosis factor; IPAQ, International Physical Activity Questionnaire. *Average HAQ value, mean (SD), is the cumulative HAQ calculated as the mean of DAS28-ESR during follow-up.

**FIGURE 2:**
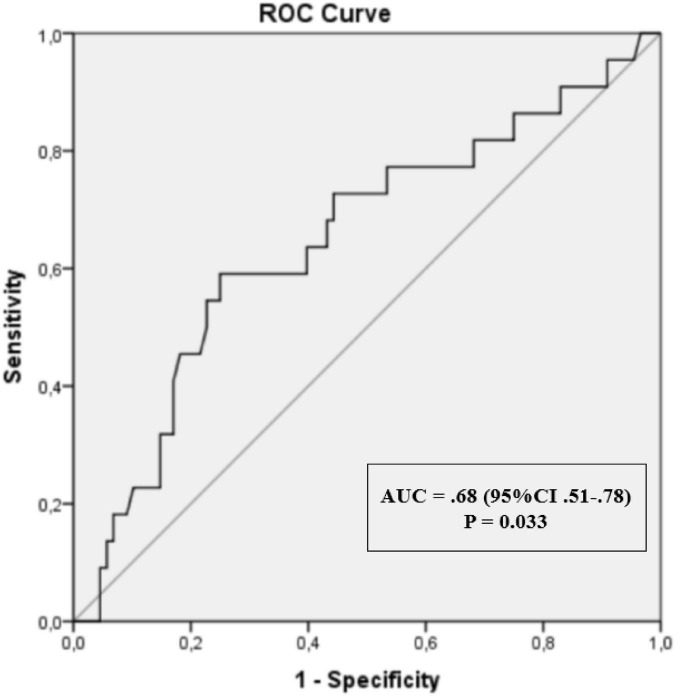
Receiver operating characteristic (ROC) curves with area under the curve (AUC) for cumulative inflammatory activity (DAS28-ESR).

ROC analysis showed that the AUC for cumulative inflammatory activity (DAS28-ESR) as a predictor for LORA was 0.68. The optimal cut-off point for cumulative inflammatory activity DAS28-ESR was 2.9 with 68% sensitivity and 62% specificity (Figure 2).

### Factors associated with inflammatory activity in patients with late-onset rheumatoid arthritis

As shown in Supplementary Table 5, of the 22 patients with LORA, 13 (59.1%) had high inflammatory activity. In addition, these patients were more frequently smokers (*p* = 0.040) and more frequently likely to have a sedentary lifestyle (*p* = 0.047), high ACPA titers (*p* = 0.040), and poorer physical function according to the mean during follow-up HAQ score (*p* = 0.005). They also had higher ESR values (*p* = 0.021) and CRP values (*p* = 0.041).

Finally, Table 4 shows the results of the multivariate logistic regression analysis for the dependent variable high inflammatory activity in patients with LORA. In this group, high ACPA titers and poorer physical function (HAQ) were associated with a greater risk of inflammatory activity. Once again, physical activity proved to be protective.

**TABLE 4:** Multivariate analysis of factors associated with cumulative inflammatory activity in patients with late-onset rheumatoid arthritis.

Variable	Univariate OR (95% CI %)	Multivariate OR (95% CI %)	*P*-value
Age in years	1.773 (0.574–1.040)		
Female sex	1.800 (0.308–10.517)		
Smoking (active/past)	2.681 (0.767–9.373)		
High ACPA (> 340 μ/mL)	5.333 (1.892–39.619)	4.598 (1.307–37.880)	0.032
Presence of erosions	2.667 (0.423–16.826)		
Average HAQ*	7.187 (1.183–23.676)	7.419 (1.071–16.179)	0.043
Body mass index (kg/m^2^)	0.963 (0.817–1.134)		
IL-6, pg/mL	1.020 (0.960–1.120)		
Il-1β, pg/mL	0.486 (0.100–1.994)		
TNF, pg/mL	1.296 (0.990–1.994)		
IPAQ, METs	0.998 (0.995–0.998)	0.998 (0.996–0.999)	0.007

The dependent variable is high inflammatory activity (DAS28-ESR ≥ 3.2). Nagelkerke *R*^2^ = 0.398. Variables included in the equation: sex, age, smoking history, high ACPA titer (> 340 μ/mL), HAQ (METS). ACPA, anti–citrullinated peptide antibody; HAQ, Health Assessment Questionnaire; IL, interleukin; CRP, C-reactive protein; TNF, tumor necrosis factor; IPAQ, International Physical Activity Questionnaire. *Average HAQ value, mean (SD), is the cumulative HAQ calculated as the mean of DAS28-ESR during follow-up.

## Discussion

Previous studies have found that clinical presentation, severity, and prognosis of RA can vary according to age of onset (2, 3, 5–7). However, the question of whether inflammatory activity over time is greater in older patients has not been addressed. In line with the objectives of the present study, we found that patients with LORA had higher cumulative activity than patients with YORA. In fact, late onset was the factor most closely associated with cumulative activity. Several studies have reported that patients with LORA seem to have more acute joint involvement, with more systemic inflammation and poorer physical function (4, 6, 14, 26).

Several authors have tried to explain why these differences arise in patients with LORA. Some point to selection bias, because physicians prefer less intensive treatments in patients with LORA than in younger patients owing to the greater frequency of comorbid conditions and risk of infection in this group (27). In this sense, data from the register of biologic therapy of the British Society of Rheumatology show that younger patients more frequently received bDMARDs than older patients (28). Another potential explanation is that older patients had a more proinflammatory immune system, with higher levels of proinflammatory cytokines than patients with YORA, possibly owing to the more marked activation of CD4 T cells and M1 macrophages that is typical of older age and contributes to greater tissue damage and persistence of the inflammatory response (29, 30). Abdelmagid et al. (29) report higher levels of inflammatory cytokines (IL-1ß, IL-6, and CRP) in patients with LORA; a positive correlation was observed between these cytokines and DAS28-ESR. Given these possible explanations, the use of DMARDs (both csDMARDS and bDMARDs) by patients with LORA did not differ from that of other patients, although they did have higher levels of CRP and IL-6.

In addition to LORA, other factors associated with cumulative activity included smoking, high ACPA titers, and greater deterioration of physical function during the disease course. Physical activity had a protective effect. The HAQ is the most commonly used questionnaire in RA (31), and poorer physical function according to this instrument has also been associated with greater inflammatory activity in patients with RA (32–34). The presence of ACPA has been associated with greater inflammatory activity and severity and with the development of erosions and deformity (35–37). ACPA is involved in the immunopathogenesis of the disease through formation of neutrophil extracellular traps, modulation of Fc receptors, and the functions of monocytes, osteoclasts, and osteoblasts (35, 38). Furthermore, smoking is the environmental factor most closely related to onset of RA. Smokers, especially those with the shared epitope, are particularly prone to developing ACPA and, therefore, carry a high risk of RA. Current or past smoking has been reported to favor a systemic proinflammatory state (39–41).

Similarly, the factors associated with greater inflammatory activity in patients with LORA in our study included high ACPA titers, poorer physical function, and physical activity. It remains unclear whether immunosenescence in RA is primary or secondary to inflammatory activity (42). However, aging of the immune system contributes to more marked impairment of the control mechanisms in innate and acquired immunity, with increased production of autoantibodies and other proinflammatory functions in patients with LORA (4). However, our multivariate analyses revealed no association between smoking and inflammatory activity in LORA. This inconsistency could be due to the fact that the sample was too small to detect an association. Nevertheless, environmental and genetic interactions could differ between LORA and other types of RA (4). In fact, Gonzalez-Gay et al. (43) showed that YORA was strongly associated with DRB1*04, unlike LORA, which was associated with DRB1*01. Therefore, different combinations of environmental and genetic factors could contribute to the development of LORA and to inflammation in patients with LORA. In addition, both sedentary lifestyle and disability are more frequently associated with sarcopenia and increased inflammatory activity in patients with LORA (44–46).

We found comorbid conditions to be more prevalent in patients with LORA than in older healthy controls and patients with YORA. This finding is consistent with those of other studies, where, in addition to the associated risk of RA itself, the effect of old age on the immune system could play a role in LORA (4, 47).

Our study is subject to a series of limitations. First, one of the limitations of the study is the cross-sectional evaluation of the levels of inflammatory cytokines. However, the patients were from a prospective early stage RA cohort in which all data on inflammation were collected longitudinally according to a predesigned protocol. In fact, a strength of our study is that it is the only one to date to evaluate the association between LORA and cumulative inflammatory activity during the disease course. As reported elsewhere (48, 49), severity can be better assessed based on cumulative activity over time than on activity at a specific time point. On the other hand, another limitation of our study is that patients with RA have received immunomodulators and their impact on the levels of cytokines and inflammatory activity at index date may be affected, however, this bias is operating in all patients and is minor because the management we do in our Medical Unit is very adjusted to the recommendations of the clinical practice guidelines, so the treatments are very homogeneous. In addition, the low number of cases with LORA (22 patients) may hamper identification of some differences between groups. However, we were able to detect differences in inflammatory activity between groups of patients and fulfill the objectives of our study.

In conclusion, we found that disease onset was at ≥ 60 years in 20% of patients with RA and that late onset was the factor most closely associated with greater cumulative inflammatory burden over time. Other factors, such as high ACPA titers and impaired physical function, were associated with greater cumulative inflammatory activity, both in all patients with RA and in those with LORA. Patients with LORA had more comorbid conditions than healthy controls and other patients. Therefore, in patients with LORA, inflammatory activity and comorbid conditions should be closely followed.

## Data availability statement

The original contributions presented in the study are included in the article/Supplementary material, further inquiries can be directed to the corresponding author.

## Ethics statement

The studies involving human participants were reviewed and approved by the Local Medical Ethics Committee, Hospital Regional Universitario de Malaga (Project identification code 4/2016, P19). The patients/participants provided their written informed consent to participate in this study.

## Author contributions

N-MV and JL-M: conceptualization, investigation, visualization, and writing—original draft preparation. N-MV and A-FN: methodology, formal analysis, and supervision. JL-M and AM: investigation and writing—original draft preparation. R-RR, SM-A, AG-S, FO-M, JR, and LC-G: conceptualization, data curation, methodology, formal analysis, supervision, and writing—review and editing. All authors have read and agreed to the published version of the manuscript.

## Footnotes

1
https://www.who.int/news-room/fact-sheets/detail/ageing-and-health

## Abbreviations

RA: rheumatoid arthritis
LORA: late-onset rheumatoid arthritis
YORA: young-onset rheumatoid arthritis
BMI: body mass index
RF: rheumatoid factor
ACPA: anti–citrullinated peptide antibody
DMARD: disease-modifying antirheumatic drug
csDMARD: conventional synthetic DMARD
bDMARD: biologic DMARD
DAS28: 28-joint Disease Activity Score
HAQ: Health Assessment Questionnaire
ESR: erythrocyte sedimentation rate
IL: interleukin
CRP: C-reactive protein
TNF: tumor necrosis factor
IGF: insulin-like growth factor
IPAQ: International Physical Activity Questionnaire
MEDAS: Validated questionnaire to determine adherence to a Mediterranean diet.
