# Supplementary material for: Inflammatory profile of incident cases of late-onset compared with young-onset rheumatoid arthritis: A nested cohort study
Source: Front Med (Lausanne). 2022 Nov 8;9:1016159. doi: 10.3389/fmed.2022.1016159 (PMC9679221; doi:10.3389/fmed.2022.1016159)
Supplement: Supplementary file 1 [file Data_Sheet_1.docx]

Supplementary table 1: Clinical-epidemiologic characteristics of patients with RA and controls by age

|  | LORA  N= 22 | YORA  N=88 | Controls ≥60  N=41 | P Value RA ≥60 vs. RA <60 | P Value onset of RA ≥60 vs. Controls ≥60 |
| --- | --- | --- | --- | --- | --- |
| Epidemiologic characteristics |  |  |  |  |  |
| Age in years, mean (SD) | 69.2 (4.6) | 52.5 (6.3) | 67.4 (5.0) | <0.001 | 0.158 |
| Female sex; n (%) | 14 (63.6) | 74 (84.1) | 34 (82.0) | 0.032 | 0.122 |
| Caucasian race, n (%) | 22 (100.0) | 86 (97.7) | 41 (100.0) | 0.475 | 1.000 |
| BMI (kg/m^2^), mean (SD) | 29.8 (5.3) | 28.0 (5.0) | 28.0 (4.2) | 0.151 | 0.145 |
| Smoking history |  |  |  | 0.690 | 0.016 |
| Never smoked, n (%) | 10 (45.5) | 37 (42.0) | 33 (80.5) |  |  |
| Ex-smoker, n (%) | 7 (31.8) | 23 (26.1) | 4 (9.8) |  |  |
| Active smoker, n (%) | 5 (22.7) | 28 (31.8) | 4 (9.8) |  |  |
| Comorbidities |  |  |  |  |  |
| Arterial hypertension, n (%) | 11 (50.0) | 17 (19.3) | 15 (36.6) | 0.003 | 0.303 |
| Diabetes mellitus, n (%) | 2 (9.1) | 4 (4.5) | 1 (2.4) | 0.401 | 0.237 |
| Dyslipidemia, n (%) | 8 (36.4) | 17 (19.3) | 13 (31.7) | 0.088 | 0.709 |
| Obesity (WHO) (BMI ≥30), n (%) | 10 (45.5) | 28 (31.8) | 11 (26.8) | 0.229 | 0.113 |
| Cardiovascular disease, n (%) | 6 (27.3) | 12 (13.6) | 3 (7.3) | 0.122 | 0.031 |
| Sjögren syndrome, n (%) | 3 (13.6) | 12 (13.6) | 0 (0.0) | 1.000 | <0.001 |
| Osteoporosis, n (%) | 7 (31.8) | 12 (13.6) | 6 (14.6) | 0.044 | 0.048 |
| Clinical characteristics |  |  |  |  |  |
| Time since diagnosis of RA, months, median (IQR) | 83.8 (76.3-101.9) | 97.1 (78.1-129.2) | - | 0.072 | NR |
| Diagnostic delay, months, median (IQR) | 6.8 (4.6-11.3) | 8.1 (4.5-13.0) | - | 0.782 | NR |
| Erosions, n (%) | 15 (68.2) | 53 (60.2) | - | 0.492 | NR |
| RF >10, n (%) | 18 (81.8) | 72 (81.0) | 0 (0.0) | 1.000 | <0.001 |
| ACPA >20 U/ml, n (%) | 18 (81.8) | 70 (79.5) | 0 (0.0) | 0.812 | <0.001 |
| High ACPA >340 U/mL, n (%) | 8 (36.4) | 28 (31.8) | 0 (0.0) | 0.684 | <0.001 |
| Treatment |  |  |  |  |  |
| csDMARD, n (%) | 20 (87.0) | 82 (88.2) | - | 0.873 | NR |
| Methotrexate, n (%) | 16 (69.6) | 63 (67.7) | - | 0.867 | NR |
| Leflunomide, n (%) | 3 (13.0) | 9 (9.7) | - | 0.635 | NR |
| Sulfasalazine, n (%) | 2 (8.7) | 11 (11.8) | - | 0.670 | NR |
| Hydroxychloroquine, n (%) | 0 (0.0) | 9 (9.7) | - | 0.126 | NR |
| bDMARD, n (%) | 6 (26.1) | 36 (38.7) | - | 0.259 | NR |
| Anti-TNF-α, n (%) | 5 (21.7) | 27 (29.0) | - | 0.483 | NR |
| Anti-IL-6, n (%) | 1 (4.3) | 6 (6.5) | - | 0.704 | NR |
| Rituximab, n (%) | 0 (0.0) | 2 (2.2) | - | 0.664 | NR |
| Tofacitinib, n (%) | 0 (0.0) | 1 (1.1) | - | 0.175 | NR |
| Combination of csDMARD and bDMARD, n (%) | 4 (17.4) | 30 (32.3) | - | 0.124 | NR |
| Corticosteroids at cut-off, n (%) | 3 (13.0) | 17 (18.3) | - | 0.552 | NR |

*Abbreviations: LORA: late-onset rheumatoid arthritis; YORA: young-onset rheumatoid arthritis; SD: standard deviation; IQR, interquartile range; BMI: body mass index; RF: rheumatoid factor; ACPA: anti–citrullinated peptide antibody; DMARD: disease-modifying antirheumatic drug; csDMARD: conventional synthetic DMARD; bDMARD, biologic DMARD.*

Supplementary table 2: Comorbidities included in the Charlson index

|  | | LORA  N= 22 | | YORA  N=88 | | Controls ≥60  N=41 | | P Value RA ≥60 vs. RA <60 | | P Value RA ≥60 vs. Controls ≥60 | |
| --- | --- | --- | --- | --- | --- | --- | --- | --- | --- | --- | --- |
| Connective tissue disease, n (%) | | 22 (100.0) | | 88 (100.0) | | 0 (0.0) | | 1.000 | | <0.001 | |
| Sjögren syndrome, n (%) | | 3 (13.6) | | 12 (13.6) | | 0 (0.0) | | 1.000 | | <0.001 | |
| Thyroid disease, n (%) | | 2 (9.1) | | 9 (10.2) | | 5 (12.0) | | 0.874 | | 0.729 | |
| Chronic obstructive pulmonary disease, n (%) | | 1 (4.5) | | 2 (2.3) | | 1 (2.4) | | 0.558 | | 0,649 | |
| Congestive heart failure, n (%) | | 3 (13.6) | | 4 (4.5) | | 3 (7.3) | | 0.118 | | 0.415 | |
| Peptic ulcer, n (%) | | 1 (4.5) | | 2 (2.3) | | 0 (0.0) | | 0.558 | | 0.169 | |
| Cardiovascular disease, n (%) | | 6 (27.3) | | 12 (13.6) | |  | |  | |  | |
| Peripheral venous disease, n (%) | | 6 (27.3) | | 14 (15.9) | | 12 (29.3) | | 0.216 | | 0.867 | |
| Diffuse interstitial lung disease, n (%) | |  | |  | |  | |  | |  | |
| Fibromyalgia syndrome, n (%) | | 1 (4.5) | | 6 (6.8) | | 2 (4.9) | | 0.696 | | 0.953 | |
| Mild liver disease, n (%) | | 1 (4.5) | | 0 (0.0) | | 0 (0.0) | | 0.045 | | 0.169 | |
| Moderate-severe kidney disease, n (%) | | 1 (4.5) | | 0 (0.0) | | 0 (0.0) | | 0.045 | | 0.169 | |
| Anxiety-depression syndrome, n (%) | | 5 (22.7) | | 12 (13.6) | | 10 (24.0) | | 0.291 | | 0.721 | |
| Cancer, n (%) | | 5 (22.7) | | 0 (0.0) | | 2 (4.9) | | <0.001 | | 0.032 | |

*Abbreviations: LORA: late-onset rheumatoid arthritis; YORA: young-onset rheumatoid arthritis.*

Supplementary table 3: Inflammatory factors and cytokines in patients with RA according to age and controls

|  | LORA  N=22 | YORA  N=88 | Controls≥60  N=41 | P Value LORA vs. YORA | P Value LORA vs. Controls ≥60 |
| --- | --- | --- | --- | --- | --- |
| Inflammatory activity |  |  |  |  |  |
| DAS28 average value, mean (SD)* | 3.3 (1.0) | 2.8 (1.0) | - | 0.033 | NR |
| Remission-low activity, n (%) | 9 (40.9) | 62 (70.5) | - | 0.010 | NR |
| Moderate-high activity, n (%) | 13 (59.1) | 26 (29.5) | - | 0.010 | NR |
| DAS28 at index-date, mean (SD) | 3.1 (0.6) | 2.9 (0.7) | - | 0.078 | NR |
| Remission-low activity, n (%) | 9 (40.9) | 60 (68.2) | - | 0.018 | NR |
| Moderate-high activity, n (%) | 13 (59.1) | 28 (31.8) | - | 0.018 | NR |
| Average HAQ value, mean (SD)** | 0.80 (0.7) | 0.71 (0.4) | - | 0.529 | NR |
| HAQ at index date, mean (SD) | 0.79 (0.6) | 0.76 (0.5) | - | 0.729 | NR |
| Laboratory parameters |  |  |  |  |  |
| ESR, mm/h, median (IQR) | 19.5 (8.7-27.5) | 13.0 (6.7-19.2) | 12.0 (6.5-17.0) | 0.102 | 0.039 |
| Hemoglobin, g/dL, median (IQR) | 13.1 (12.2-14.1) | 13.9 (13.0-14.7) | 14.0 (12.9-14.9) | 0.034 | 0.840 |
| Leukocytes, 10^9^/L, median (IQR) | 6,735 (5,780-8,407) | 6,295 (5150-8147) | 5,600 (4570-7505) | 0.258 | 0.021 |
| Platelets, 10^9^/L, median (IQR) | 236,000(200,000-260,250) | 243,000 (200,500-284,500) | 226,000(197,500-257,000) | 0.239 | 0.521 |
| Creatinine, mg/dL, median (IQR) | 0.8 (0.7-0.9) | 0.7 (0.6-0.8) | 0.6 (0.5-0.8) | 0.003 | 0.097 |
| Total cholesterol (mg/dL), mean (SD) | 208.9 (38.3) | 199.0 (37.2) | 223.1 (34.8) | 0.271 | 0.123 |
| LDL cholesterol (mg/dL), median (IQR) | 119.0 (108.3-141.7) | 113.0 (95.2-135.0) | 137.8 (110.5-155.5) | 0.222 | 0.136 |
| HDL cholesterol (mg/dL), median (IQR) | 57.5 (50.5-65.2) | 59.5 (49.2-69.0) | 61.0 (54.5-72.0) | 0.208 | 0.112 |
| Triglycerides (mg/dL), median (IQR) | 108.0 (74.5-219.0) | 84.5 (68.2-118.7) | 108.0 (81.0-146.5) | 0.025 | 0.708 |
| Homocysteine, mg/L, median (IQR) | 16.3 (7.0-34.0) | 13.9 (11.6-16.0) | 12.0 (10.1-16.3) | 0.228 | 0.025 |
| Cytokines |  |  |  |  |  |
| IL6, pg/mL, median (IQR) | 16.3 (7.0-34.0) | 9.9 (5.2-17.6) | 4.9 (3.1-7.2) | 0.045 | <0.001 |
| CRP, mg/L, mean (SD) | 6.9 (4.1) | 4.1 (3.7) | 3.1 (1.1) | 0.039 | 0.010 |
| IL-1β, pg/mL, median (IQR) | 4.3 (4.2-4.5) | 4.3 (4.1-4.4) | 2.7 (2.6-2.9) | 0.660 | <0.001 |
| TNF-α, pg/mL, median (IQR) | 6.1 (3.6-22.4) | 5.0 (3.7-22.4) | 4.1 (3.2-5.0) | 0.308 | 0.032 |
| IGF-1, pg/mL, median (IQR) | 109.0 (70.5-64.5) | 182.5 (113.8-244.0) | 58.0 (27.0-224.2) | 0.112 | 0.320 |
| LDL oxidase (U/L), median (IQR) | 1.7 (0.4-5.3) | 2.6 (0.7-5.7) | 0.8 (0.2-1.5) | 0.605 | 0.246 |
| Physical activity and Mediterranean diet |  |  |  |  |  |
| IPAQ, METs, median (IQR) | 210 (165.0-922.5) | 353.3 (198.0-890.7) | 604.0 (214.0-990.0) | 0.224 | 0.038 |
| Sedentary lifestyle, n (%) | 15 (68.2) | 50 (56.8) | 18 (43.9) | 0.430 | 0.041 |
| MEDAS (>9), n (%) | 10 (45.5) | 56 (63.6) | 26 (63.4) | 0.119 | 0.170 |

*Abbreviations: LORA: late-onset rheumatoid arthritis; YORA: young-onset rheumatoid arthritis; DAS28: 28-joint Disease Activity Score; HAQ: Health Assessment Questionnaire; ESR: erythrocyte sedimentation rate; HDL: high-density lipoprotein; LDL: low-density lipoprotein; IL: interleukin; CRP: C-reactive protein; TNF: tumor necrosis factor; IGF: insulin-like growth factor; IPAQ: International Physical Activity Questionnaire; MEDAS: Validated questionnaire to determine adherence to a Mediterranean diet. * DAS28 average value, mean (SD): is the cumulative inflammatory activity calculated as the mean of DAS28-ESR during follow-up. .** Average HAQ value, mean (SD): is the cumulative HAQ calculated as the mean of DAS28-ESR during follow-up.*

Supplementary table 4: Clinical-laboratory characteristics and cytokine profile associated with inflammatory activity in patients with rheumatoid arthritis

| Variable | High inflammatory activity (n=39) | | Low inflammatory activity (n=71) | | P Value | |
| --- | --- | --- | --- | --- | --- | --- |
| Epidemiologic characteristics |  | |  | |  | |
| Age, years, mean (SD) | 58.6 (10.2) | | 54.1 (11.1) | | 0.046 | |
| LORA (≥60 years), n (%) | 13 (33.3) | | 9 (12.7) | | 0.010 | |
| Female sex, n (%) | 29 (74.4) | | 59 (83.1) | | 0.273 | |
| Caucasian race, n (%) | 39 (100.0) | | 69 (97.2) | | 0.290 | |
| BMI (kg/m^2^), mean (SD) | 30.0 (5.8) | | 27.5 (4.4) | | 0.020 | |
| Smoking history |  | |  | | 0.009 | |
| Never smoked, n (%) | 9 (23.1) | | 38 (53.5) | |  | |
| Exsmoker, n (%) | 15 (38.5) | | 15 (21.1) | |  | |
| Active smoker, n (%) | 15 (38.5) | | 18 (25.4) | |  | |
| Comorbidities |  | |  | |  | |
| Arterial hypertension, n (%) | 13 (33.3) | | 15 (21.1) | | 0.160 | |
| Diabetes mellitus, n (%) | 4 (10.3) | | 2 (2.8) | | 0.100 | |
| Dyslipidemia, n (%) | 14 (35.9) | | 11 (15.5) | | 0.035 | |
| Obesity WHO (BMI≥30), n (%) | 19 (48.7) | 21 (29.6) | | 0.048 | |  |
| Cardiovascular disease, n (%) | 9 (23.1) | 9 (12.6) | | 0.739 | |  |
| Sjögren syndrome, n (%) | 7 (17.9) | 8 (11.3) | | 0.329 | |  |
| Osteoporosis, n (%) | 10 (25.7) | | 9 (12.7) | | 0.046 | |
| Clinical characteristics |  | |  | |  | |
| Time since diagnosis of RA, months, median (IQR) | 90.5 (79.2-109.7) | | 98.6 (77.6-113.9) | | 0.210 | |
| Diagnostic delay, months, median (IQR) | 8.9 (4.6-17.6) | | 8.1 (5.7-13.3) | | 0.983 | |
| Erosions, n (%) | 26 (66.7) | | 42 (59.2) | | 0.438 | |
| RF >10, n (%) | 33 (84.6) | | 57 (80.3) | | 0.573 | |
| ACPA >20 U/mL, n (%) | 31 (79.5) | | 57 (80.3) | | 0.921 | |
| High ACPA >340 U/mL, n (%) | 20 (51.3) | | 16 (22.5) | | 0.002 | |
| Average HAQ value, median (IQR) | 0.8 (0.3-1.1) | | 0.5 (0.2-0.9) | | 0.041 | |
| HAQ at index-date, median (IQR) | 1.0 (0.6-1.3) | | 0.5 (0.0-1.0) | | 0.004 | |
| Treatment |  | |  | |  | |
| csDMARD, n (%) | 36 (92.3) | | 66 (93.0) | | 0.900 | |
| Methotrexate, n (%) | 29 (74.4) | | 50 (70.4) | | 0.661 | |
| Leflunomide, n (%) | 3 (7.7) | | 9 (12.7) | | 0.423 | |
| Sulfasalazine, n (%) | 7 (17.9) | | 6 (8.5) | | 0.140 | |
| Hydroxychloroquine, n (%) | 2 (5.1) | | 7 (9.9) | | 0.386 | |
| bDMARD, n (%) | 17 (43.6) | | 25 (35.2) | | 0.387 | |
| Anti-TNF-α, n (%) | 13 (33.3) | | 19 (26.8) | | 0.468 | |
| Anti-IL-6, n (%) | 2 (5.1) | | 5 (7.6) | | 0.694 | |
| Rituximab, n (%) | 1 (2.6) | | 1 (1.4) | | 0.664 | |
| Tofacitinib, n (%) | 1 (2.6) | | 0 (0.0) | | 0.175 | |
| Combination of csDMARD and bDMARD, n (%) | 14 (35.9) | | 20 (28.2) | | 0.401 | |
| Corticosteroids at cut-off, n (%) | 8 (20.5) | | 12 (16.9) | | 0.639 | |
| Laboratory parameters |  | |  | |  | |
| ESR, mm/h, median (IQR) | 18.0 (10.0-26.0) | | 11.1 (7.5-18.0) | | 0.019 | |
| Hemoglobin g/dL, median (IQR) | 14.0 (12.9-14.7) | | 13.0 (12.2-13.7) | | 0.021 | |
| Leukocytes 10^9^/L, median (IQR) | 6,450 (5,440-8,900) | | 6,350 (5,340-8,000) | | 0.578 | |
| Platelets, 10^9^/L, median (IQR) | 243,000 (194,000-200,000) | | 238,000(202,000-283,000) | | 0.722 | |
| Creatinine mg/dL, median (IQR) | 0.7 (0.6-0.8) | | 0.7 (0.6-0.8) | | 0.151 | |
| Total cholesterol (mg/dL), mean (SD) | 210.5 (36.9) | | 195.8 (37.0) | | 0.050 | |
| LDL cholesterol (mg/dL), median (IQR) | 123.0 (107.0-150.0) | | 110.0 (95.5-129.0) | | 0.025 | |
| HDL cholesterol (mg/dL), median (IQR) | 58.0 (51.0-65.0) | | 60.0 (49.0-69.0) | | 0.274 | |
| Triglycerides (mg/dL), median (IQR) | 106.0 (85.0-132.0) | | 77.0 (66.0-118.0) | | 0.034 | |
| Homocysteine mg/L, median (IQR) | 14.6 (13.1-16.6) | | 13.5 (10.7-16.6) | | 0.063 | |
| Cytokines |  | |  | |  | |
| IL-6, pg/mL, median (IQR) | 12.3 (6.6-19.9) | | 8.6 (5.4-16.0) | | 0.046 | |
| CRP, mg/L, mean (SD) | 8.1 (3.9) | | 4.2 (2.3) | | 0.027 | |
| IL-1β, pg/mL, median (IQR) | 4.6 (4.2-4.5) | | 4.0 (3.9-4.4) | | 0.300 | |
| TNF-α, pg/mL, median (IQR) | 5.8 (3.4-75.2) | | 4.9 (3.7-10.2) | | 0.107 | |
| IGF-1, pg/mL, median (IQR) | 164.8 (98.5-237.5) | | 177.0 (95.4-236.4) | | 0.916 | |
| LDL oxidase (U/L), median (IQR) | 2.1 (0.9-8.8) | | 2.5 (0.5-5.5) | | 0.994 | |
| Physical activity and Mediterranean diet |  | |  | |  | |
| IPAQ, METs, median (IQR) | 198.0 (165.0-330.0) | | 495.0 (290.0-990.0) | | 0.009 | |
| Sedentary lifestyle, n (%) | 32 (82.1) | | 42 (59.2) | | 0.014 | |
| MEDAS (>9), median (IQR) | 8.0 (7.0-9.0) | | 9.0 (8.0-10.0) | | 0.023 | |

*Abbreviations: LORA: late-onset rheumatoid arthritis; BMI: body mass index; RF: rheumatoid arthritis; ACPA: anti–citrullinated peptide antibody; HAQ: Health Assessment Questionnaire; DMARD: disease-modifying antirheumatic drug; csDMARD: conventional synthetic DMARD; bDMARD: biologic DMARD; ESR: erythrocyte sedimentation rate; LDL: low-density lipoprotein; HDL: high-density lipoprotein; IL: interleukin 6; CRP: C-reactive protein; TNF: tumor necrosis factor;* *IGF: insulin-like growth factor; IPAQ: International Physical Activity Questionnaire; MEDAS: validated survey on adherence to a Mediterranean diet.*

Supplementary table 5: Clinical-laboratory characteristics and cytokine profile associated with inflammatory activity in patients with LORA

| Variable | High inflammatory activity (n=13) | | Low inflammatory activity (n=9) | | P Value | |
| --- | --- | --- | --- | --- | --- | --- |
| Epidemiologic characteristics |  | |  | |  | |
| Age in years, mean (SD) | 71.0 (3.2) | | 68.3 (5.1) | | 0.145 | |
| Female sex; n (%) | 8 (61.5) | | 6 (66.7) | | 0.806 | |
| Caucasian race, n (%) | 13 (100.0) | | 9 (10.0) | | 1.000 | |
| BMI (kg/m^2^), mean (SD) | 29.9 (5.8) | | 29.7 (5.0) | | 0.918 | |
| Smoking history |  | |  | | 0.040 | |
| Never smoked, n (%) | 3 (23.1) | | 7 (77.8) | |  | |
| Ex-smoker, n (%) | 6 (46.2) | | 1 (11.1) | |  | |
| Active smoker, n (%) | 4 (30.8) | | 1 (11.1) | |  | |
| Comorbidities |  | |  | |  | |
| Arterial hypertension, n (%) | 6 (46.2) | | 5 (55.6) | | 0.693 | |
| Diabetes mellitus, n (%) | 1 (7.7) | | 1 (11.1) | | 0.784 | |
| Dyslipidemia, n (%) | 6 (46.2) | | 2 (22.2) | | 0.251 | |
| Obesity (WHO) (BMI ≥30), n (%) | 8 (61.5) | 4 (44.4) | | 0.361 | |  |
| Cardiovascular disease, n (%) | 3 (23.1) | 3 (33.3) | | 0.595 | |  |
| Sjögren syndrome, n (%) | 1 (7.7) | 2 (22.2) | | 0.329 | |  |
| Osteoporosis, n (%) | 5 (38.5) | | 2 (22.2) | | 0.421 | |
| Clinical characteristics |  | |  | |  | |
| Time since diagnosis of RA, months, median (IQR) | 79.6 (64.8-107.4) | | 89.7 (77.4-101.9) | | 0.794 | |
| Diagnostic delay, months, median (IQR) | 6.8 (4.6-16.6) | | 6.0 (4.8-20.0) | | 0.845 | |
| Erosions, n (%) | 9 (69.4) | | 6 (66.7) | | 0.694 | |
| RF >10, n (%) | 10 (76.9) | | 8 (88.9) | | 0.474 | |
| ACPA >20 U/mL, n (%) | 10 (76.9) | | 8 (88.9) | | 0.474 | |
| High ACPA >340 U/mL, n (%) | 7 (53.8) | | 1 (11.1) | | 0.040 | |
| HAQ average value, mean (SD) | 0.9 (0.4) | | 0.4 (0.2) | | 0.005 | |
| HAQ at index-date, mean (SD) | 0.9 (0.5) | | 0.5 (0.5) | | 0.185 | |
| Treatment |  | |  | |  | |
| csDMARD, n (%) | 11 (84.6) | | 9 (100.0) | | 0.217 | |
| Methotrexate, n (%) | 9 (69.2) | | 7 (77.8) | | 0.658 | |
| Leflunomide, n (%) | 2 (15.4) | | 1 (11.1) | | 0.774 | |
| Sulfasalazine, n (%) | 1 (7.7) | | 1 (11.1) | | 0.784 | |
| Hydroxychloroquine, n (%) | 0 (0.0) | | 0 (0.0) | | 1.000 | |
| bDMARD, n (%) | 4 (30.8) | | 2 (22.2) | | 0.658 | |
| Anti-TNF-α, n (%) | 3 (23.1) | | 2 (22.2) | | 0.962 | |
| Anti-IL-6, n (%) | 1 (7.7) | | 0 (0.0) | | 0.394 | |
| Rituximab, n (%) | 0 (0.0) | | 0 (0.0) | | 1.000 | |
| Tofacitinib, n (%) | 0 (0.0) | | 0 (0.0) | | 1.000 | |
| Combination of csDMARD and bDMARD, n (%) | 2 (15.4) | | 2 (22.2) | | 0.683 | |
| Corticosteroids at cut-off, n (%) | 1 (7.7) | | 2 (22.2) | | 0.329 | |
| Laboratory parameters |  | |  | |  | |
| ESR, mm/h, median (IQR) | 22.0 (14.0-36.0) | | 10.0 (8.0-12.0) | | 0.021 | |
| Hemoglobin, g/dL, median (SD) | 13.6 (12.8-14.1) | | 14.6 (13.3-15.4) | | 0.082 | |
| Leukocytes, 10^9^/L, median (SD) | 7,660 (5,735-9,606) | | 6,550 (5,780-7,355) | | 0.471 | |
| Platelets, 10^9^/L, median (SD) | 245,000(214,500-277,500) | | 219,000(179,000-251,000) | | 0.235 | |
| Creatinine, mg/dL, median (SD) | 0.8 (0.6-0.9) | | 0.7 (0.7-0.8) | | 0.896 | |
| Total cholesterol (mg/dL), mean (SD) | 219.3 (35.5) | | 189.6 (35.3) | | 0.096 | |
| LDL cholesterol (mg/dL), median (IQR) | 127.0 (112.4-162.5) | | 110.0 (96.2-123.5) | | 0.051 | |
| HDL cholesterol (mg/dL), median (IQR) | 58.0 (51.5-62.5) | | 57.0 (46.5-66.8) | | 0.896 | |
| Triglycerides (mg/dL), median (IQR) | 110.0 (90.0-159.5) | | 75.0 (64.0-220.0) | | 0.471 | |
| Homocysteine, median (IQR) | 16.0 (13.7-17.8) | | 12.6 (12.0-14.0) | | 0.067 | |
| Cytokines |  | |  | |  | |
| IL-6, pg/mL, median (IQR) | 16.3 (7.0-34.0) | | 13.0 (6.9-40.6) | | 0.111 | |
| CRP, mg/L, mean (SD) | 8.8 (6.0) | | 3.2 (1.1) | | 0.041 | |
| IL-1β, pg/mL, median (IQR) | 4.6 (4.0-5.1) | | 3.9 (3.6-4.6) | | 0.167 | |
| TNF-α, pg/mL, median (IQR) | 6.4 (3.7-16.6) | | 4.1 (3.5-8.0) | | 0.143 | |
| IGF-1, pg/mL, median (IQR) | 155.1 (79.3-234.2) | | 88.5 (65.5-142.9) | | 0.093 | |
| LDL oxidase (U/L), median (IQR) | 1.7 (0.7-8.7) | | 1.4 (0.2-5.1) | | 0.852 | |
| Physical activity and Mediterranean diet |  | |  | |  | |
| METs, median (IQR) | 200.0 (165.0-920.0) | | 900.0 (165.0-990.0) | | 0.002 | |
| Sedentary lifestyle, n (%) | 11 (84.6) | | 4 (44.4) | | 0.047 | |
| MEDAS (>9), n (%) | 5 (38.5) | | 5 (55.6) | | 0.209 | |

*Abbreviations: RA: rheumatoid arthritis; BMI: body mass index; RF: rheumatoid factor; ACPA: anti–citrullinated peptide antibody; HAQ: Health Assessment Questionnaire; DMARD: disease-modifying antirheumatic drug; csDMARD: conventional synthetic DMARD; bDMARD: biologic DMARD; ESR: erythrocyte sedimentation rate; LDL: low-density lipoprotein; HDL: high-density lipoprotein; IL: interleukin; CRP: C-reactive protein; TNF: tumor necrosis factor; IGF: insulin-like growth factor; MEDAS: Validated questionnaire on adherence to a Mediterranean diet.*
